# Epidemiological investigations of diarrhea in children in Praia city, Cape Verde

**DOI:** 10.3389/fmicb.2022.1059431

**Published:** 2022-12-22

**Authors:** Denise Andrade Colito, Roberto Dorta-Guerra, Hailton Spencer Da Costa Lima, Carine Pina, Deisy Gonçalves, Basilio Valladares, Pilar Foronda

**Affiliations:** ^1^Faculty of Science and Technology, University of Cape Verde, Palmarejo, Cape Verde; ^2^Departamento de Matemáticas, Estadística e IO, Universidad de La Laguna, San Cristóbal de La Laguna, Spain; ^3^Instituto Universitario de Enfermedades Tropicales y Salud Pública de Canarias, Universidad de La Laguna, San Cristóbal de La Laguna, Spain; ^4^Departamento de Obstetricia y Ginecología, Pediatría, Medicina Preventiva y Salud Pública, Toxicología, Medicina Legal y Forense y Parasitología, Universidad de La Laguna, San Cristóbal de La Laguna, Spain

**Keywords:** diarrhea, bacteria, virus, risk factors, Cape Verde, children

## Abstract

**Introduction:**

Diarrheal disease is a major cause of infant mortality and morbidity in Africa and results primarily from contaminated food and water sources, but its prevalence predictors in Cape Verde are not completely known. For this reason, this study aimed to identify the etiological agents of diarrhea in Cape Verdean children and assess its associated risk factors.

**Methods:**

A survey questionnaire was used, and a total of 105 stool samples from children with diarrhea aged 0–12 years at the Central Hospital of Praia (Santiago, Cape Verde) were analyzed. The analyses were carried out using Biofire FilmArray Gastrointestinal Panels. Possible risk factors for these pathogens were analyzed using logistic regression, chi-square tests, or Fisher’s exact test.

**Results:**

Among the bacteria, enteroaggregative *Escherichia coli* (45.71%; 95% CI: 36.71–56.70), enteropathogenic *E. coli* (40%; 95% CI: 30.56–50.02), Shigella/enteroinvasive *E. coli* (29.52%; 95% CI: 21.02–39.22), *E. coli* enterotoxigenic (12.38%; 95% CI: 6.76–20.24), *Campylobacter* sp. (10.48%; 95% CI: 5.35–1.97), *Vibrio* sp. (4.76%; 95% CI: 1.56–10.76), *Clostridioides difficile* (3.81%; 95% CI: 1.05–9.47), *Vibrio cholerae* (2.86%; 0.59–8.12), Shiga-like toxin-producing *E. coli* (2.86%; 0.59–8.12) and *Salmonella* sp. (0.95%; 0.02–5.19) were identified; four viruses, Rotavirus A (28.57%; 95% CI: 20.18–38.21), Sapovirus I. II. IV and V (11.43%; 95% CI: 6.05–19.11), Norovirus GI.GII (6.67%; 95% CI: 2.72–13.25) and Adenovirus F 40.41 (6.67%; 95% CI: 2.72–13.25) were also observed. All the pathogens detected in this study were found in coinfections. Significant associations with risk factors were found; specifically, having a bathroom at home reduced the risk of *Campylobacter* sp., having animals at home increased the risk of *Shigella*/EIEC infection, and drinking bottled water reduced the risk of Sapovirus infection.

**Discussion:**

From the findings of this study, it can be concluded that, in Cape Verde, there is a high prevalence and diversity of pathogens among children. Our results could help to establish an adequate diagnosis and effective treatments for diarrheal disease.

## 1 Introduction

Diarrheal disease is a major cause of infant mortality and morbidity in the world (Acácio et al., 2019) and mainly results from contaminated food and water sources (Chen et al., 2015; Laham et al., 2015; Pires et al., 2015). Infectious diarrhea is widespread in developing countries, where it remains a public health problem and has a substantially higher impact in low-income countries and regions with poor water quality, sanitation, and food security (Pires et al., 2015). In Africa, diarrhea is the leading cause of illness and death among young children, and nearly 50% of deaths from diarrhea in young children occur in Africa (Workie et al., 2019). This disease exposes children to various other infections, predisposing them to malnutrition (Workie et al., 2019), impaired physical development, and stunted growth (Laham et al., 2015).

Diarrhea can be attributed to a variety of gastrointestinal (GI) pathogens, including protozoa, viruses, and bacteria (Laham et al., 2015; Pires et al., 2015; Hawash et al., 2017), and the distribution and prevalence vary with the geographical area, due to various environmental, social, and geographical aspects.

The most common etiologic agents include bacteria such as *Campylobacter* sp., enteropathogenic *Escherichia coli* (EPEC), enterotoxigenic *E. coli* (ETEC), *Salmonella* sp. and *Shigella* sp.; viruses: rotavirus, norovirus, adenovirus and astrovirus, and protozoa; *Giardia* sp. and *Cryptosporidium* sp., and *Entamoeba histolytica* (Laham et al., 2015; Acácio et al., 2019; Saaed and Ongerth, 2019). Infections can be transmitted to humans through food or water, person-to-person contact, exposure to animals, or acquired from the environment (Hawash et al., 2017).

In Cape Verde, the predictors of the prevalence of diarrheal diseases are not fully known; however, every year, there are many cases of gastrointestinal problems in children, often of unknown causes. According to the 2018 Statistical Report of the Ministry of Health and Social Security of the Republic of Cape Verde (Msss.Relatório estatístico, 2018), diarrheal diseases have an incidence rate of 2493.4/10.000 inhabitants and 287.4/10.000 inhabitants in children under and over 5 years old, respectively. However, data on the etiology of this pathology in children in Cape Verde are scarce, and little is known about the infection intensity profile and the underlying risk factors in the country. Therefore, this study was designed to detect the different enteric pathogens that cause gastroenteritis in children in the city of Praia and associate them with possible risk factors to formulate appropriate control strategies and predict the risks posed to the communities under consideration.

## 2 Materials and methods

### 2.1 Study area

**FIGURE 1:**
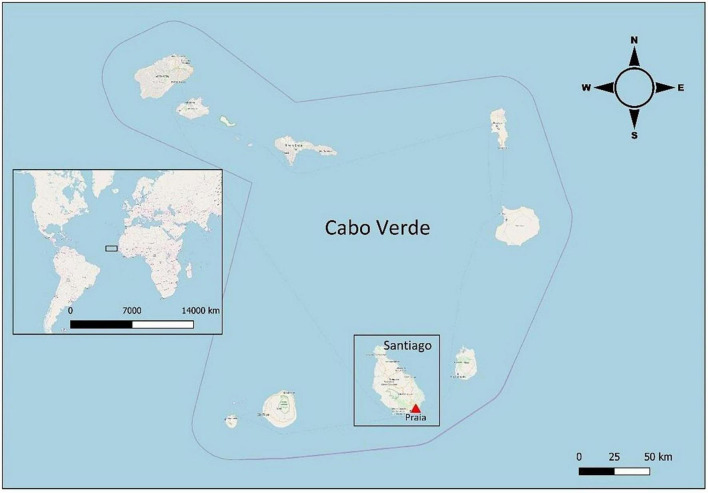
Location of Praia in Santiago Island (Cape Verde). Map modified with QGIS 3.8.0-Zanzibar (www.qgis.org) from OpenStreetMap.org. OpenStreetMap^®^ is open data, licensed under the Open Data Commons Open Database License (ODbL) by the OpenStreetMap Foundation (OSMF).

Cape Verde is a small Atlantic archipelago located between 15°20’ and 14°50’ north latitude and 23°50’ and 23°20’ west longitude. Santiago is the largest of the ten islands of the Archipelago, with a 991 Km^2^ area and a perimeter of 970 Km (Figure 1). Praia is the capital of Santiago and Cape Verde, where most of the country’s population lives.

The samples for this study were collected at the pediatric emergency and ambulatory service at Hospital Dr. Agostinho Neto (HAN) in Praia city, Santiago.

### 2.2 Study design and population

For this study, 105 fecal samples from children less than 12 years old and with diarrhea were collected from July 2018 to August 2019 and preserved in Cary Blair (Biomerieux, France) until use. Fresh stool samples were collected when children with diarrhea attended the hospital and were included in this study.

Parents/caregivers filled out a questionnaire on different variables, namely address, symptoms, age, gender, education degree, name of school/kindergarten, kind of drinking water, presence of animals at home, occupation of the parents, sanitation at home, preparation of fruits and vegetables, and antibiotic use.

### 2.3 Laboratory procedures

All the samples were molecularly analyzed with the Biofire^®^ FilmArray^®^ Gastrointestinal (GI) Panel with a Biofire^®^ FilmArray^®^ integrated system (Biomerieux, France). The FilmArray GI Panel is a multiplexed nucleic acid test intended for use with FilmArray systems for the simultaneous qualitative detection and identification of multiple gastrointestinal viral (Adenovirus F 40/41, Astrovirus, Norovirus GI/GII, Rotavirus A, and Sapovirus I, II, IV,V), bacteria [*Campylobacter* (*C. jejuni, C. coli*, and *C. upsaliensis*), *Clostridium difficile* toxin A/B, *Plesiomonas shigelloides*, *Salmonella*, *Vibrio* (*V. parahaemolyticus, V. vulnificus*, and *V. cholerae*), *Yersinia entercolitica*, enteroaggregative *E. coli* (EAEC), enteropathogenic *E. coli* (EPEC), enterotoxigenic *E. coli* (ETEC) *lt/st*, Shiga-like toxin-producing *E. coli* (STEC) *stx1/stx2*, *E. coli* O157, *Shigella*/enteroinvasive *E. coli* (EIEC)] and protozoa.

In nest multiplex PCR, the tests are performed in two stages. In the first stage, using multiple outer primers, multiplex PCRs are performed on the target template present in the sample, while in the second stage, a singleplex PCR is performed, further amplifying the DNA procured during the first PCR. The inner primers that are used in the second PCR are made of those sequences “nested” within the first PCR products, and the time taken to complete the test is <2 h (FilmArray^®^ Panels, Gastrointestinal Panel).

### 2.4 Statistical analysis

Data analyses were carried out using IBM SPSS, version 25 (IBM Corporation, Armonk, NY, USA), Microsoft Excel, and R 3.5.1 statistical software. The results are presented as means ± standard deviations (SDs) for the continuous data and proportions (prevalences) for the categorical data. For prevalence rates, 95% confidence intervals using the approximate or exact method, as appropriate, were included. A chi-square test or Fisher’s exact test, as appropriate, was performed to study the associations between the presence of parasites and some sociodemographic and hygienic variables such as sex, sample zone, scholarship, age, diarrhea per day, stool description, classification of diarrhea, water source, the existence of bathroom at home, the preparation of fruits and vegetables, and the presence of animals in the compound. The results with *p* < 0.05 were considered statistically significant.

To determine the predictor variables for the presence of viruses or bacteria, a binary logistic regression model was fitted, and the variables with a *p*-value < 0.2 during the bi-variate analysis were included in the multivariable analysis. All the assumptions for binary logistic regression were checked. Finally, the variables found to be significant in the final model (*p*-value < 0.05) were declared as predictors. The crude odds ratios (CORs) and adjusted odds ratios (AOR) were reported with 95% confidence intervals. The Omnibus Tests of Model Coefficients (*p* < 0.05) table was used to check whether the final model (with explanatory variables included) improved over the baseline model (null model).

For the coinfection statistical analysis, the data on protozoa parasites, previously published in Colito et al. (2021), were also included. These data were obtained from the same samples and with the same methodology.

### 2.5 Ethical statement

The project was approved by the National Ethical Commission for the Health Research of the Ministry of Health and Social Security of Cape Verde with reference n° 28/2018. Signed informed consent was obtained from all the parents or legal guardians of the study participants.

## 3 Results

**FIGURE 2:**
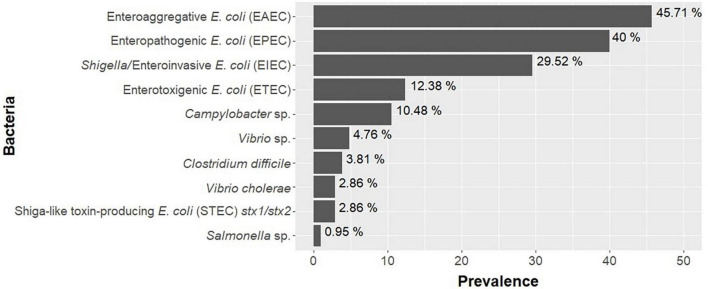
Prevalence of bacteria found in children in the study (*n* = 105) in Praia, Cape Verde.

In this study, 10 types of bacteria and 4 different viruses were identified, with a general prevalence of 70.48% (74/105; 95% CI: 60.78–78.98) and 48.57% (51/105; 95% CI: 38.70–58.53), respectively. The bacteria identified were enteroaggregative *E. coli* (EAEC) in 45.71% of the samples (48/105; 95% CI: 36.71–56.70); enteropathogenic *E. coli* (EPEC) in 40% (42/105; 95% CI: 30.56–50.02); *Shigella*/enteroinvasive *E. coli* (EIEC) in 29.52% (31/105; 95% CI: 21.02–39.22); enterotoxigenic *E. coli* (ETEC) in 12.38% (13/105; 95% CI: 6.76–20.24); *Campylobacter* sp. in 10.48% (11/105; 95% CI: 5.35–1.97); *V. parahaemolyticus*/*vulnificus*/*cholerae* at 4.76% (5/105; 95% CI: 1.56–10.76); *C. difficile* at 3.81% (4/105; 95% CI: 1.05–9.47); *V. cholerae* at 2.86% (3/105; 95% CI: 0.59–8.12); Shiga-like toxin-producing *E. coli* (STEC) in 2.86% (3/105; 95% CI: 0.59–8.12); and *Salmonella* sp. in 0.95% (1/105; 95% CI: 0.02–5.19) (Table 1 and Figure 2). No positive samples were detected for *E. coli* O157, *Plesiomonas shigelloide*, and *Yersinia enterocolitica*.

**TABLE 1:** Frequency (%) of diarrhea pathogens by sex, age, attending kindergarten or school, water to drink, bathroom at home, and animals living in the compound, from July 2018 to August 2019.

		EAEC	EPEC	EIEC	ETEC	*Camp*.	*Vibri.*	*Clost*.	*V. chol.*	STEC	*Salmon.*	Rotav.	Sapov.	Norov.	Adenov.
Prevalence	+/*n* (%)	48/105 (45,7)	42/105 (40,0)	31/105 (29,5)	13/105 (12,4)	11/105 (10,5)	5/105 (4,8)	4/105 (3,8)	3/105 (2,9)	3/105 (2,9)	1/105 (1,0)	30/105 (28,6)	12/105 (11,4)	7/105 (6,7)	7/105 (6,7)
	(95% CI)	(36.71–56.70)	(30.56–50.02)	(21.02–39.22)	(6.76–20.24)	(5.35–17.97)	(1.56–10.76)	(1.05–9.47)	(0.59–8.12)	(0.59–8.12)	(0.02–5.19)	(20.18–38.21)	(6.05–19.11)	(2.72–13.25)	(2.72–13.25)
Sex, +/*n* (%)	Male	23/53 (43,4)	20/53 (37,7)	15/53 (28,3)	5/53 (9,4)	6/53 (11,3)	3/53 (5,7)	3/53 (5,7)	2/53 (3,8)	1/53 (1,9)	0/53 (0,0)	11/53 (20,8)	5/53 (9,4)	5/53 (9,4)	2/53 (3,8)
	Female	25/51 (49,0)	22/51 (43,1)	16/51 (31,4)	8/51 (15,7)	5/51 (9,8)	2/51 (3,9)	1/51 (2,0)	1/51 (2,0)	2/51 (3,9)	1/51 (2,0)	19/51 (37,3)	7/51 (13,7)	2/51 (3,9)	5/51 (9,8)
	Sig.	n.s.	n.s.	n.s.	n.s.	n.s.	n.s.	n.s.	n.s.	n.s.	n.s.	n.s.	n.s.	n.s.	n.s.
Age, +/*n* (%)	0–30 months	39/42 (44,8)	37/42 (42,5)	24/42 (27,6)	10/42 (11,5)	8/42 (9,2)	5/42 (5,7)	4/42 (4,6)	3/42 (3,4)	0/42 (0,0)	0/42 (0,0)	28/42 (32,2)	10/42 (11,5)	6/42 (6,9)	6/42 (6,9)
	>30 months	9/18 (50,0)	5/18 (27,8)	7/18 (38,9)	3/18 (16,7)	3/18 (16,7)	0/18 (0,0)	0/18 (0,0)	0/18 (0,0)	3/18 (16,7)	1/18 (5,6)	2/18 (11,1)	2/18 (11,1)	1/18 (5,6)	1/18 (5,6)
	Sig.	n.s.	n.s.	n.s.	n.s.	n.s.	n.s.	n.s.	n.s.	0,004	n.s.	n.s.	n.s.	n.s.	n.s.
Attending kindergarten or school, +/*n* (%)	No	35/73 (47,9)	33/73 (45,2)	18/73 (24,7)	10/73 (13,7)	7/73 (9,6)	4/73 (5,5)	4/73 (5,5)	2/73 (2,7)	0/73 (0,0)	0/73 (0,0)	25/73 (34,2)	8/73 (11,0)	4/73 (5,5)	5/73 (6,8)
	Yes	13/31 (41,9)	9/31 (29,0)	13/31 (41,9)	3/31 (9,7)	4/31 (12,9)	1/31 (3,2)	0/31 (0,0)	1/31 (3,2)	3/31 (9,7)	1/31 (3,2)	5/31 (16,1)	4/31 (12,9)	3/31 (9,7)	2/31 (6,5)
	Sig.	n.s.	n.s.	n.s.	n.s.	n.s.	n.s.	n.s.	n.s.	0,025	n.s.	n.s.	n.s.	n.s.	n.s.
Water to drink, +/*n* (%)	Bottle	31/63 (49,2)	23/63 (36,5)	17/63 (27,0)	10/63 (15,9)	10/63 (15,9)	1/63 (1,6)	3/63 (4,8)	1/63 (1,6)	3/63 (4,8)	1/63 (1,6)	17/63 (27,0)	11/63 (17,5)	3/63 (4,8)	1/63 (1,6)
	Non- bottled	17/42 (40,5)	19/42 (45,2)	14/42 (33,3)	3/42 (7,1)	1/42 (2,4)	4/42 (9,5)	1/42 (2,4)	2/42 (4,8)	0/42 (0,0)	0/42 (0,0)	13/42 (31,0)	1/42 (2,4)	4/42 (9,5)	6/42 (14,3)
	Sig.	n.s.	n.s.	n.s.	n.s.	0,047	n.s.	n.s.	n.s.	n.s.	n.s.	n.s.	0,025	n.s.	0,016
Bathroom at home, +/*n* (%)	No	5/13 (38,5)	3/13 (23,1)	4/13 (30,8)	1/13 (7,7)	4/13 (30,8)	0/13 (0,0)	1/13 (7,7)	0/13 (0,0)	0/13 (0,0)	0/13 (0,0)	2/13 (15,4)	2/13 (15,4)	0/13 (0,0)	0/13 (0,0)
	Yes	43/91 (47,3)	39/91 (42,9)	27/91 (29,7)	12/91 (13,2)	7/91 (7,7)	5/91 (5,5)	3/91 (3,3)	3/91 (3,3)	3/91 (3,3)	1/91 (1,1)	28/91 (30,8)	10/91 (11,0)	7/91 (7,7)	7/91 (7,7)
	Sig.	n.s.	n.s.	n.s.	n.s.	0,030	n.s.	n.s.	n.s.	n.s.	n.s.	n.s.	n.s.	n.s.	n.s.
Animals living in the compound, +/*n* (%)	No	25/66 (37,9)	25/66 (37,9)	15/66 (22,7)	5/66 (7,6)	7/66 (10,6)	4/66 (6,1)	3/66 (4,5)	2/66 (3,0)	1/66 (1,5)	0/66 (0,0)	21/66 (31,8)	5/66 (7,6)	3/66 (4,5)	4/66 (6,1)
	Yes	22/38 (57,9)	16/38 (42,1)	16/38 (42,1)	8/38 (21,1)	4/38 (10,5)	1/38 (2,6)	1/38 (2,6)	1/38 (2,6)	2/38 (5,3)	1/38 (2,6)	9/38 (23,7)	7/38 (18,4)	4/38 (10,5)	3/38 (7,9)
	Sig.	n.s.	n.s.	n.s.	n.s.	n.s.	n.s.	n.s.	n.s.	n.s.	n.s.	n.s.	n.s.	n.s.	n.s.

EAEC, enteroaggregative *E. coli*; EPEC, enteropathogenic *E. coli*; EIEC, *Shigella*/Enteroinvasive *E. coli*; ETEC, enterotoxigenic *E. coli; Camp., Campylobacter* (*jejuni, coli, and upsaliensis*); *Vibri., Vibrio* (*parahaemolyticus, vulnificus, and cholerae*); *Clost*., clostridium difficile toxin A/B; *V. chol., Vibrio cholerae*; STEC, Shiga-like toxin-producing *E. coli; Salmon., Salmonella* sp.; Rotav., Rotavirus A; Sapov., Sapovirus I, II, IV, V; Norov., Norovirus GI/GII; Adenov., Adenovirus F 40/41; n.s., not significant.

**FIGURE 3:**
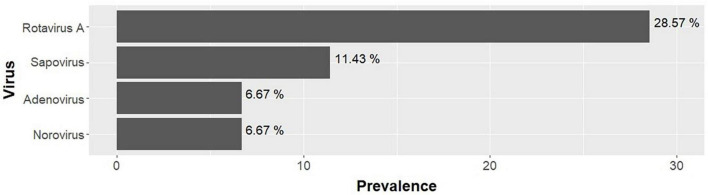
Prevalence of viruses found in children in the study (*n* = 105) in Praia, Cape Verde.

Regarding the viruses, Rotavirus A was identified in 28.57% (30/105; 95% CI: 20.18–38.21); Sapovirus I, II, IV, and V in 11.43% (12/105; 95% CI: 6.05–19.11); Norovirus GI.GII in 6.67% (7/105; 95% CI: 2.72–13.25); and Adenovirus F 40.41 in 6.67% (7/105; 95% CI: 2.72–13.25). Astrovirus was not detected in any of the samples (Figure 3).

### 3.1 Coinfection study

**FIGURE 4:**
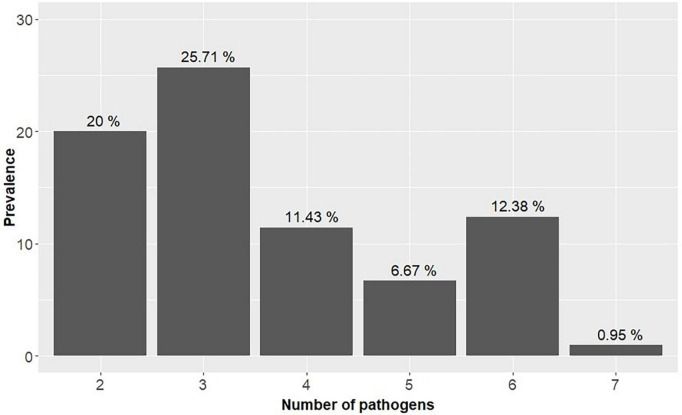
Prevalence of coinfection in children in the study (*n* = 105) in Praia, Cape Verde.

The overall coinfection rate was 77%, and the number of pathogens per child ranged from 1 to 7, with a prevalence of 13 and 0.95%, respectively. Most children harbored two and three pathogens simultaneously (Figure 4).

All the pathogens detected in this study were found in coinfections in some cases, and *Campylobacter* sp., *C. difficile*, *Salmonella* sp., *V. cholerae*, EPEC, ETEC, ECST, and Adenovirus were detected in patients only as coinfections. The coinfections between EAEC and EPEC were more frequent (28%), followed by EAEC and *G. duodenalis* (19.4%) and EAEC and EIEC (18.5%). A high level of association was also identified between enteropathogenic *E. coli* and *G. duodenalis* and between Rotavirus A and EAEC and EPEC (Table 2).

**TABLE 2:** Prevalence of association of different pathogens in cases of coinfections in children from Praia, Cape Verde.

Coinfection	+/*n* (prevalence)	CI
EAEC + EPEC	29/103 (28.2%)	19.7–37.9%
EAEC + *G. duodenalis*	20/103 (19.4%)	12.3–28.4%
EAEC + EIEC	19/103 (18.4%)	11.5–27.3%
EIEC + *G. duodenalis*	16/105 (15.2%)	9.0–23.6%
EAEC + Rotavirus	16/103 (15.5%)	9.1–24.0%
EPEC + Rotavirus	15/105 (14.3%)	8.2–22.5%
EPEC + *G. duodenalis*	15/105 (14.3%)	8.2–22.5%
EPEC + EIEC	14/105 (13.3%)	7.5–21.4%
Rotavirus A + *G. duodenalis*	11/105 (10.5%)	5.3–18.0%
EAEC + ETEC	10/103 (9.7%)	4.8–17.1%
EAEC + EPEC + EIEC	11/103 (10.7%)	5.5–18.3%
EAEC + EPEC + *G. duodenalis*	10/103 (9.7%)	4,8–17.1%
EAEC + EIEC + *G. duodenalis*	10/103 (9.7%)	4.8–17.1%
EAEC + EPEC + Rotavirus A	9/103 (8.7%)	4.1–15.9%
EAEC + ETEC + EIEC	8/103 (7.8%)	3.4–14.7%

+, number of children with the coinfection; *n*, number of children analyzed; CI, confidence interval.

### 3.2 Risk factors for the presence of pathogens

To determine whether sociodemographic factors were associated with the presence of pathogens, the proportion of children with each potential risk factor was compared in the presence or absence of a pathogen group. The bivariable analysis revealed that age, attending kindergarten or school, the source of drinking water, the presence of a bathroom at home, and the presence of animals in the compound were the variables (*p*-value < 0.2) associated with at least one of the pathogens.

From the factors tested in the current study, only “bathroom at home” was significantly associated with the presence of *Campylobacter* sp. in the final model (*p* = 0.020); children with a bathroom at home had 81.0% reduced adjusted odds ratios of the presence of *Campylobacter* (AOR: 0.19, 95% CI: 0.05, 0.77), compared with those with no bathroom at home. On the other hand, of the factors tested, only “animals in home” was significantly associated with the presence of *Shigella*/EIEC in the final model (*p* = 0.040); children with animals in the compound had a 2.42-fold (AOR: 2.42, 95% CI: 1.02, 5.76) increased adjusted odds ratios for the presence of *Shigella* compared with those with no animals in the compound. “Water to drink” was associated with the presence of Sapovirus (*p* = 0.043) and Adenovirus (*p* = 0.034) in the final model. Children drinking bottled water had a 10.33-fold (AOR: 10.33, 95% CI: 1.20, 89.29) increase adjusted odds ratios for the presence of Adenovirus and 88.8% reduced adjusted odds ratios for the presence of Sapovirus (AOR: 0.11, 95% CI: 0.01, 0.91) compared with those drinking non-bottled water (see Table 3).

**TABLE 3:** Correlations between sociodemographic factors and presence of pathogens.

		+/*n* (%)	COR (95% CI)	*P*-value	AOR (95% CI)
***Campylobacter***					
Water to drink	Non-bottled water	10/63 (15.9)			
	Bottled water	1/42 (2.4)	0.13 (0.02–1.05)	0.056	–
Bathroom at home	No	4/13 (30.8)			
	Yes	7/91 (7.7)	0.19 (0.05–0.77)	0.020	0.19 (0.05–0.77)*
***Shigella*/enteroinvasive *E. coli***					
Kindergarten or school	Do not go to kindergarten or school	18/73 (24.7)			
	Attend kindergarten or school	13/31 (41.9)	2.21 (0.91–5.37)	0.081	–
Animals living in the compound	No	15/66 (22.7)			
	Yes	16/38 (42.1)	2.47 (1.04–5.87)	0.040	2.42 (1.02–5.76)*
**Sapovirus**					
Water to drink	Non-bottled water	11/63 (17.5)			
	Bottled water	1/42 (2.4)	0.12 (0.01–0.93)	0.043	0.11 (0.01–0.91)*
Animals living in the compound	No	5/66 (7.6)			
	Yes	7/38 (18.4)	2.76 (0.81–9.39)	0.105	–
**Adenovirus**					
Water to drink	Non-bottled water	1/63 (1.6)			
	Bottled water	6/42 (14.3)	10.33 (1.20–89.29)	0.034	10.33 (1.20–89.29)*

COR, crude odds ratio; AOR, adjusted odds ratio; OR, odds ratio; CI, confidence interval.

*Significant at *p*-value < 0.05.

## 4 Discussion

This study is the first to investigate the intestinal pathogens that affect children in Cape Verde, including bacteria and viruses, and many of the identified pathogens were detected for the first time in the country. A high prevalence of infection by pathogens was detected in the children participating in the study (70.48 and 48.57%, respectively), with 77% of the children having two or more pathogens in their stools, and some children with up to seven pathogens in the same sample. In this context, coinfection is commonly reported in other parts of Africa where intestinal pathogens are endemic (Mbae et al., 2013; Patzi-Vargas et al., 2015; Shrestha et al., 2018).

The high prevalence of intestinal pathogens was also reported in other similar studies. In a study carried out on children from Angola, bacteria and viruses were detected in 78 and 50% of the samples of feces, respectively (Pelkonen et al., 2018), and in Sudan, 48% of samples tested positive for diarrheagenic *Escherichia coli* and 22% for Rotavirus A (Saeed et al., 2015). In this study, EAEC, EPEC, and EIEC were the predominant pathogens detected in the analyzed samples. These bacteria are among the most common bacterial causes of morbidity and mortality in children worldwide (Lozer et al., 2013; Saka et al., 2019) and a major public health challenge in developing countries (Saka et al., 2019), including Cape Verde. They are not routinely screened, and the ability to detect them is limited in Africa (Odetoyin et al., 2016). For this reason, diarrheagenic *E. coli* infection is often underdiagnosed during routine microbiological analyses, especially in localized areas with limited resources (Saka et al., 2019).

On the other hand, *Campylobacter* sp., *Salmonella* sp., and *Shigella* sp. are the best-known pathogens that cause bacterial gastroenteritis in the world (Shah et al., 2016; Chlebicz and Śliżewska, 2018), but in the present study, *Campylobacter* sp., and particularly *Salmonella* sp., were identified with low prevalence rates of 10.48 and 0.95%, respectively. In the present study, the risk factor associated with the presence of *Campylobacter* sp. was the presence of a bathroom at home. The authors found that the main risk factors for *Campylobacter* sp. is the exposure to an unsanitary environment and the consumption of contaminated food and water (Asuming-Bediako, 2019). The lack of a bathroom at home can promote the spread of the infection since the exposure of feces to the environment can lead to the contamination of food and/or water (Zenebe et al., 2020), and when water treatment is inefficient, it can lead to the spread of the infection.

Cholera is a notifiable disease in Cape Verde, caused by the strains of the bacterium *V. cholerae* (mainly serogroups O1 and O139) (Mohammed et al., 2018; Connor et al., 2019). In the present study, *V. cholerae* was detected in 2.86% of the children, and although the disease has not been confirmed, the presence of this bacterium is of concern. Improved sanitation and access to safe water have largely eliminated cholera in high-income countries, but it remains a problem in low-income countries (Ali et al., 2015), where adequate sanitation and clean water are not widely available, and large epidemics can occur (Connor et al., 2019).

Regarding virus infection, the present study detected the presence of at least one of the four viruses identified in about 50% of the children with diarrhea. The high prevalence of viral infection can potentially lead to the mismanagement of acute viral gastroenteritis (antibiotic treatment) due to the lack of adequate diagnostic tools for acute viral gastroenteritis in health facilities in Cape Verde, which, in turn, can contribute to the increase in antimicrobial resistance in the country. This is the first study carried out in Cape Verde involving viral detection in stool samples, and most of the identified viruses were detected for the first time in the country.

Rotavirus A infections are reported to be the leading cause of severe acute gastroenteritis in young children and infants worldwide (Gupta et al., 2019; Damtie et al., 2020; Waure et al., 2020); however, in Cape Verde, vaccination against RVA is not included in the national vaccination calendar. The results for RVA infection obtained in this study are in line with those reported in different countries, mainly in poor or developing countries; for example, in a study carried out in Taiwan, RVA remained the main cause of viral gastroenteritis that requires hospitalization in children, even after vaccine implementation, but at a much lower rate (43 and 46–21.2%) (Chen et al., 2015). In India, RVA was the most prevalent virus (54.9%) from 2009 to 2015, followed by NoV (25.7%), Astrovirus (8.3%), HAdV (4.9%), and SaV (0.7%) (Gupta et al., 2018). In our study, SaV was more prevalent than NoV, which contradicts those studies that detected NoV at a higher rate of infection (Grytdal et al., 2016; Gelaw et al., 2019; Oliveira-Tozetto et al., 2021; Rossouw et al., 2021). The overall rate of HAdV infection in children with diarrhea observed in this study was 6.67%, similar to those reported in other countries such as Thailand (7.2%) (Kumthip et al., 2020), Korea (6.5%) (Kim et al., 2017), the Republic of Congo (10.5%) (Medkour et al., 2020), India (11.8%) (Banerjee et al., 2017), and Bangladesh (10.7%) (Afrad et al., 2018), but there are also reports from other regions with higher prevalence rates, such as China (28.94%) (Qiu et al., 2018), Ethiopia (32%) (Gelaw et al., 2019), and Gabon (19.6%) (Lekana-Douki et al., 2015).

The risk factors associated with the presence of AdV and SaV in children were the “type of drinking water” for both and the “presence of animals in the home” for SaV; importantly, children who drank bottled water were less infected. Other studies also observed a significant association between the positive cases of Sapovirus and sources of drinking water (municipal tap water, borehole, river, and spring), a fact explained by the poor microbial quality of piped water (tap water) in a low socioeconomic environment and high level of indicator microorganism counts in water storage containers compared with indoor tap water (Magwalivha et al., 2018).

The high prevalence of coinfections in this study (77%) shows that a multipathogenic etiology of diarrhea is common in the study population. Coinfections with enteropathogens often increase the severity of diarrhea, exacerbating the outcome of the infection in humans (Zhang et al., 2016; Vergadi et al., 2021), some enteropathogens have synergism, and the pathogenic potential of each organism seems to be increased during coinfection (Zhang et al., 2016). In the present study, the coinfections between EAEC and EPEC, as well as EAEC and *G. duodenalis*, were more prevalent, and all pathogens were found in coinfections. A study conducted on East African children observed positive associations for *Campylobacter* and ETEC, *Campylobacter* and *Cryptosporidium*, *Shigella* and EPEC, and for *Shigella* and EPEC, and suggested that these combinations could potentiate symptoms (Andersson et al., 2018). Moyo et al. (2017) verified significant positive interactions between Rotavirus and *Giardia* and between Norovirus GII and EAEC in a multiplicative model, while Bhavnani et al. (2012) in turn found that simultaneous infection with Rotavirus and *Giardia* or Rotavirus and *E. coli* (including *Shigellae*) resulted in a greater risk of having diarrhea than would be expected if the coinfecting organisms acted independently of each other.

From the findings of this study, it can be concluded that, despite the efforts to improve the quality of water and sanitation and the implementation of the mass deworming program in children, infections by intestinal pathogens transmitted through water and food continue to prevail in Cape Verde. This is because the detected pathogens are related to the precarious conditions of sanitation, hygiene, and quality of drinking water, with the fecal–oral route being the main means of transmission. For this reason, it is necessary to establish programs to monitor the quality of drinking water in Cape Verde. This work also indicates the need to implement appropriate diagnostic methods for the detected pathogens in hospitals and health centers, thus allowing the application of an effective treatment to prevent the mortality and morbidity associated with different species of pathogens.

## Data availability statement

The original contributions presented in this study are included in the article/supplementary material, further inquiries can be directed to the corresponding author.

## Ethics statement

The studies involving human participants were reviewed and approved by National Ethical Commission for Health Research of the Ministry of Health and Social Security of Cape Verde. Written informed consent to participate in this study was provided by the participants’ legal guardian/next of kin.

## Author contributions

DC, HD, CP, and DG collected the samples and patients data. DC, HD, and PF analyzed the samples. RD-G carried out the statistical analyses. BV and PF obtained the funding and supervised the work. DC, RD-G, and PF did the main writing of the manuscript. All authors have read and approved the final manuscript.

## References

[B1] Acácio S. Mandomando I. Nhampossa T. Quintó L. Vubil D. Sacoor C. (2019). Risk factors for death among children 0-59 months of age with moderate-to-severe diarrhea in Manhiça district, Southern Mozambique. *BMC Infect. Dis.* 19:322. 10.1186/s12879-019-3948-9 30987589 PMC6466733

[B2] Afrad M. Avzun T. Haque J. Haque W. Hossain M. Rahman A. (2018). Detection of enteric- and non-enteric adenoviruses in gastroenteritis patients, Bangladesh, 2012-2015. *J. Med. Virol.* 90 677–684. 10.1002/jmv.25008 29244212

[B3] Ali M. Nelson A. Lopez A. Sack D. (2015). Updated global burden of cholera in endemic countries. *PLoS Negl. Trop. Dis.* 9:e0003832. 10.1371/journal.pntd.0003832 26043000 PMC4455997

[B4] Andersson M. Kabayiza J. Elfving K. Nilsson S. Msellem M. Andreas M. (2018). Coinfection with enteric pathogens in East African children with acute gastroenteritis — associations and interpretations. *Am. J. Trop. Med. Hyg.* 98 1566–1570. 10.4269/ajtmh.17-0473 29692296 PMC6086148

[B5] Asuming-Bediako N. (2019). Campylobacter at the human – food interface: The African perspective. *Pathogens* 8:87. 10.3390/pathogens8020087 31242594 PMC6631673

[B6] Banerjee A. De P. Manna B. Chawla-Sarkar M. (2017). Molecular characterization of enteric adenovirus genotypes 40 and 41 identified in children with acute gastroenteritis in Kolkata, India during 2013–2014. *J. Med. Virol.* 89 606–614. 10.1002/jmv.24672 27584661

[B7] Bhavnani D. Goldstick J. Cevallos W. Trueba G. Eisenberg J. (2012). Synergistic effects between rotavirus and coinfecting pathogens on diarrheal disease: Evidence from a community-based study in Northwestern Ecuador. *Am. J. Epidemiol.* 176 387–395. 10.1093/aje/kws220 22842722 PMC3499114

[B8] Chen C. Wu F. Huang Y. Chang W. Wu H. Wu C. (2015). Clinical and epidemiologic features of severe viral gastroenteritis in children: A 3-year surveillance, multicentered study in Taiwan with partial rotavirus immunization. *Medicine* 94:e1372. 10.1097/MD.0000000000001372 26287425 PMC4616446

[B9] Chlebicz A. Śliżewska K. (2018). Campylobacteriosis, salmonellosis, yersiniosis, and listeriosis as zoonotic foodborne diseases: A review. *Int. J. Environ. Res. Public Health* 15:863. 10.3390/ijerph15050863 29701663 PMC5981902

[B10] Colito D. Dorta-Guerra R. Da Costa Lima H. Pina C. Gonsalvez D. Valladares B. (2021). Intestinal parasites among children with diarrhoea from Santiago (Cape Verde). *Arch Dis. Child* 106 828–830. 10.1136/archdischild-2020-319978 33451993

[B11] Connor B. Dawood R. Riddle M. Hamer D. (2019). Cholera in travellers:A systematic review. *J. Travel Med.* 26:taz085. 10.1093/jtm/taz085 31804684 PMC6927393

[B12] Damtie D. Melku M. Tessema B. Vlasova A. (2020). Prevalence and genetic diversity of rotaviruses among under-five children in Ethiopia: A systematic review and meta-analysis. *Viruses* 12:62. 10.3390/v12010062 31947826 PMC7019712

[B13] Gelaw A. Pietsch C. Mann P. Liebert U. (2019). Molecular detection and characterisation of sapoviruses and noroviruses in outpatient children with diarrhoea in Northwest Ethiopia. *Epidemiol. Infect.* 147:e218. 10.1017/S0950268819001031 31364546 PMC6625200

[B14] Grytdal S. DeBess E. Lee L. Blythe D. Ryan P. Biggs C. (2016). Incidence of norovirus and other viral pathogens that cause acute gastroenteritis (AGE) among kaiser permanente member populations in the United States, 2012-2013. *PLoS One* 11:e0148395. 10.1371/journal.pone.0148395 27115485 PMC4846013

[B15] Gupta S. Chaudhary S. Bubber P. Ray P. (2019). Epidemiology and genetic diversity of group a rotavirus in acute diarrhea patients in pre-vaccination era in Himachal Pradesh. India. *Vaccine* 37 5350–5356. 10.1016/j.vaccine.2019.07.037 31331769

[B16] Gupta S. Krishnan A. Sharma S. Kumar P. Aneja S. Ray P. (2018). Changing pattern of prevalence, genetic diversity, and mixed infections of viruses associated with acute gastroenteritis in pediatric patients in New Delhi. India. *J. Med. Virol.* 90 469–476. 10.1002/jmv.24980 29064572

[B17] Hawash Y. Ismail K. Almehmadi M. (2017). High frequency of enteric protozoan, viral, and bacterial potential pathogens in community-acquired acute diarrheal episodes: Evidence based on results of luminex gastrointestinal pathogen panel assay. *Korean J. Parasitol.* 55 513–521. 10.3347/kjp.2017.55.5.513 29103266 PMC5678467

[B18] Kim J. Lee S. Ko D. Hyun J. Kim H. Song W. (2017). Associations of adenovirus genotypes in Korean acute gastroenteritis patients with respiratory symptoms and intussusception. *Biomed. Res. Int.* 2017:1602054. 10.1155/2017/1602054 28255553 PMC5309414

[B19] Kumthip K. Khamrin P. Ushijima H. Chen L. Li S. Maneekarn N. (2020). Genetic recombination and diversity of sapovirus in pediatric patients with acute gastroenteritis in Thailand, 2010-2018. *PeerJ* 8:e8520. 10.7717/peerj.8520 32071820 PMC7007980

[B20] Laham N. Elyazji M. Al-Haddad R. Ridwan F. (2015). Prevalence of enteric pathogen-associated community gastroenteritis among kindergarten children in Gaza. *J. Biomed. Res.* 29 61–68. 10.7555/JBR.29.20130108 25745477 PMC4342437

[B21] Lekana-Douki S. Kombila-Koumavor C. Nkoghe D. Drosten C. Drexler J. Leroy E. (2015). Molecular epidemiology of enteric viruses and genotyping of rotavirus A, adenovirus and astrovirus among children under 5 years old in Gabon. *Int. J. Infect. Dis.* 34 90–95. 10.1016/j.ijid.2015.03.009 25796432

[B22] Lozer D. Souza T. Monfardini M. V. Vicentini F. Kitagawa S. Scaletsky I. (2013). Genotypic and phenotypic analysis of diarrheagenic *Escherichia coli* strains isolated from Brazilian children living in low socioeconomic level communities. *BMC Infect. Dis.* 13:418. 10.1186/1471-2334-13-418 24010735 PMC3846636

[B23] Magwalivha M. Kabue J. Traore A. Potgieter N. (2018). Prevalence of human sapovirus in low and middle income countries. *Adv. Virol.* 2018:5986549. 10.1155/2018/5986549 30245718 PMC6139206

[B24] Mbae C. Nokes D. Mulinge E. Nyambura J. Waruru A. Kariuki S. (2013). Intestinal parasitic infections in children presenting with diarrhoea in outpatient and inpatient settings in an informal settlement of Nairobi, Kenya. *BMC Infect. Dis.* 13:243. 10.1186/1471-2334-13-243 23705776 PMC3673844

[B25] Medkour H. Amona I. Akiana J. Davoust B. Bitam I. (2020). Adenovirus infections in African humans and wild non-human primates: Great diversity and cross-species transmission. *Viruses* 12:657. 10.3390/v12060657 32570742 PMC7354429

[B26] Mohammed Y. Aboderin A. Okeke I. Olayinka A. (2018). Antimicrobial resistance of *Vibrio cholerae* from sub-Saharan Africa: A systematic review. *Afr. J. Lab. Med.* 7:778. 10.4102/ajlm.v7i2.778 30643734 PMC6325272

[B27] Moyo S. Kommedal Ø Blomberg B. Hanevik K. Tellevik M. Maselle S. (2017). Comprehensive analysis of prevalence, epidemiologic characteristics, and clinical characteristics of monoinfection and coinfection in diarrheal diseases in children in Tanzania. *Am. J. Epidemiol.* 186 1074–1083. 10.1093/aje/kwx173 28541454 PMC5860328

[B28] Msss. Relatório estatístico (2018). *Ministério da saúde e segurança soc da república cabo verde [Internet].* 115. Available online at: https://minsaude.gov.cv/wpfd_file/relatorio-estatistico-2018-final/ (accessed May, 2019).

[B29] Odetoyin B. Hofmann J. Aboderin A. Okeke I. (2016). Diarrhoeagenic *Escherichia coli* in mother-child Pairs in Ile-Ife, South Western Nigeria. *BMC Infect. Dis.* 16:28. 10.1186/s12879-016-1365-x 26809819 PMC4727348

[B30] Oliveira-Tozetto S. Santiso-Bellón C. Ferrer-Chirivella J. Navarro-Lleó N. Vila-Vicent S. Rodríguez-Díaz J. (2021). Epidemiological and genetic characterization of sapovirus in patients with acute gastroenteritis in Valencia (Spain). *Viruses* 13:184. 10.3390/v13020184 33530573 PMC7911121

[B31] Patzi-Vargas S. Zaidi M. Perez-Martinez I. León–Cen M. Michel-Ayala A. Chaussabel D. (2015). Diarrheagenic *Escherichia coli* carrying supplementary virulence genes are an important cause of moderate to severe diarrhoeal disease in Mexico. *PLoS Negl. Trop. Dis.* 9:e0003510. 10.1371/journal.pntd.0003510 25738580 PMC4349884

[B32] Pelkonen T. Dos Santos M. Roine I. Dos Anjos E. Freitas C. Peltola H. (2018). Potential diarrheal pathogens common also in healthy children in Angola. *Pediatr. Infect. Dis. J.* 37 424–428. 10.1097/INF.0000000000001781 28885460 PMC5916461

[B33] Pires S. Fischer-Walker C. Lanata C. Devleesschauwer B. Hall A. Kirk M. (2015). Aetiology-specific estimates of the global and regional incidence and mortality of diarrhoeal diseases commonly transmitted through food. *PLoS One* 10:e0142927. 10.1371/journal.pone.0142927 26632843 PMC4668836

[B34] Qiu F. Shen X. Li G. Zhao L. Chen C. Duan S. (2018). Adenovirus associated with acute diarrhea: A case-control study. *BMC Infect. Dis.* 18:450. 10.1186/s12879-018-3340-1 30176819 PMC6122197

[B35] Rossouw E. Brauer M. Meyer P. du Plessis N. Avenant T. Mans J. (2021). Virus etiology, diversity and clinical characteristics in south african children hospitalised with gastroenteritis. *Viruses* 13:215. 10.3390/v13020215 33573340 PMC7911269

[B36] Saaed F. Ongerth J. (2019). Giardia and cryptosporidium in children with diarrhea, Kufra, Libya, a North African migration route city. *Int. J. Hyg. Environ. Health* 222 840–846. 10.1016/j.ijheh.2019.04.006 31085111

[B37] Saeed A. Abd H. Sandstrom G. (2015). Microbial aetiology of acute diarrhoea in children under five years of age in Khartoum, Sudan. *J. Med. Microbiol.* 64 432–437. 10.1099/jmm.0.000043 25713206 PMC4635512

[B38] Saka H. Dabo N. Muhammad B. García-Soto S. Ugarte-Ruiz M. Alvarez J. (2019). Diarrheagenic *Escherichia coli* pathotypes from children younger than 5 years in Kano State, Nigeria. *Front. Public Heal.* 7:348. 10.3389/fpubh.2019.00348 31828054 PMC6890574

[B39] Shah M. Kathiiko C. Wada A. Odoyo E. Bundi M. Miringu G. (2016). Prevalence, seasonal variation, and antibiotic resistance pattern of enteric bacterial pathogens among hospitalized diarrheic children in suburban regions of central Kenya. *Trop. Med. Health* 44:39. 10.1186/s41182-016-0038-1 27942243 PMC5126808

[B40] Shrestha A. Schindler C. Odermatt P. Gerold J. Erismann S. Sharma S. (2018). Intestinal parasite infections and associated risk factors among schoolchildren in Dolakha and Ramechhap districts, Nepal: A cross-sectional study. *Parasit. Vectors* 11:532. 10.1186/s13071-018-3105-0 30268160 PMC6162948

[B41] Vergadi E. Maraki S. Dardamani E. Ladomenou F. (2021). Polymicrobial gastroenteritis in children. *Acta Paediatr.* 110 2240–2250. 10.1111/apa.15854 33755990

[B42] Waure C. Sarnari L. Chiavarini M. Ianiro G. Monini M. Alunno A. (2020). 10-year rotavirus infection surveillance: Epidemiological trends in the pediatric population of Perugia province. *Int. J. Environ. Res. Public Health* 17:1008. 10.3390/ijerph17031008 32033439 PMC7036783

[B43] Workie G. Akalu T. Baraki A. (2019). Environmental factors affecting childhood diarrheal disease among under-five children in jamma district, South Wello zone, Northeast Ethiopia. *BMC Infect. Dis.* 19:804. 10.1186/s12879-019-4445-x 31519160 PMC6743097

[B44] Zenebe T. Zegeye N. Eguale T. (2020). Prevalence of Campylobacter species in human, animal and food of animal origin and their antimicrobial susceptibility in Ethiopia: A systematic review and meta-analysis. *Ann. Clin. Microbiol. Antimicrob.* 19:61. 10.1186/s12941-020-00405-8 33302968 PMC7731538

[B45] Zhang S. Zhou Y. Xu W. Tian L. Chen J. Chen S. (2016). Impact of co-infections with enteric pathogens on children suffering from acute diarrhea in Southwest China. *Infect. Dis. Poverty* 5:64. 10.1186/s40249-016-0157-2 27349521 PMC4922062

